# Chlorogenic acid alters ileal microbiota and metabolites in broiler chickens under immune stress

**DOI:** 10.1128/spectrum.03312-24

**Published:** 2025-06-12

**Authors:** Ziwei Wang, Wenrui Zhen, Yi Zhang, Caifang Guo, Xiaodie Zhao, Penghui Ma, Koichi ito, Bingkun Zhang, Cai Zhang, Dongying Bai, Yanbo Ma

**Affiliations:** 1 Department of Animal Physiology, College of Animal Science and Technology, Henan University of Science and Technology 74623, Luoyang, China; 2 Henan International Joint Laboratory of Animal Welfare and Health Breeding, College of Animal Science and Technology, Henan University of Science and Technology, Luoyang, China; 3 Department of Food and Physiological Models, Graduate School of Agricultural and Life Sciences, The University of Tokyo, Ibaraki, Japan; 4 State Key Laboratory of Animal Nutrition, Department of Animal Nutrition and Feed Science, College of Animal Science and Technology, China Agricultural University 630135 , Beijing, China; 5 Innovative Research Team of Livestock Intelligent Breeding and Equipment, Science & Technology Innovation Center for Completed Set Equipment, Longmen Laboratory, Luoyang, China; Jilin University, Changchun, China

**Keywords:** chlorogenic acid, immune stress, gut microbiota, gut metabolites, broilers

## Abstract

Immune stress in broilers can cause severe economic losses, and chlorogenic acid (CGA) is an effective plant extract for alleviating immune stress. This study investigated the effects of CGA on the intestinal microbiota and metabolites of broilers under immune stress. A group of 312 broiler chicks was randomly divided into four treatment groups: Saline control, lipopolysaccharide (LPS), LPS + CGA (LCGA), and Saline + CGA (SCGA). The SCGA and LCGA groups were fed a basal diet supplemented with 1,000 mg/kg CGA throughout the whole experimental period. The LPS and LCGA groups were injected with 0.5 mg/kg LPS on days 14, 15, and 16 to induce immune stress. At day 17, the ileal contents were collected for analysis. Results showed that LPS-induced immune stress decreased the abundance of *Subdoligranulum*, thereby lowering microbial richness. However, dietary CGA increased beneficial bacteria like *Clostridiaceae* (*P* < 0.05) and *Candidatus Arthromitus* (*P* < 0.01) in immune-stressed broilers, while reducing harmful *Lachnospiraceae* (*P* < 0.05) and *Desulfovibrionaceae* (*P* < 0.05). Non-targeted metabolomics revealed that immune stress significantly increased methylmalonic acid, N-acetyl-L-aspartic acid, trans-ferulic acid, and pantothenic acid, while significantly downregulating pyroglutamic acid, L-glutamic gamma-semialdehyde, and biliverdin, indicating that immune stress (LPS) induced inflammation and oxidative damage. CGA addition reduced corticosterone, prostaglandin B2, trans-ferulic acid, and methylmalonic acid levels, while increasing pyroglutamic acid, L-glutamic gamma-semialdehyde, biliverdin, chenodeoxycholic acid, desmosterol, and 4-hydroxycinnamic acid, confirming that CGA can alleviate inflammation and enhance antioxidant capacity by influencing ileal microbiota and specific metabolite synthesis.

**IMPORTANCE:**

Our previous research indicated that CGA could effectively alleviate immune stress in broilers. However, it was unclear whether its antistress effects were achieved by altering the gut microbiota and metabolites in the ileum of immune-stressed broilers. In this study, CGA altered gut microbiota and metabolites associated with alleviating immune stress were provided, which will provide new insights into strategies to target gut microbiota and metabolites to relieve immune stress in broilers. These findings contribute to a better understanding of the mechanism of action of CGA on the attenuation of immune stress and provide new approaches to immune stress therapy by regulating gut microbiota and metabolites.

## INTRODUCTION

Broiler chickens are particularly vulnerable to exogenous stimuli during the intensive breeding process. These exogenous stimuli can induce immune stress in the animals and compromise their welfare (1, 2). Immune stress can also lead to a decline in the activity of antioxidant enzymes and the capacity to scavenge free radicals in the intestines of broilers, severely damaging intestinal health (3). A large amount of free radicals and other reactive oxygen species (ROS) are produced in the intestinal tract of broilers under immune stress, which can further aggravate damage to the intestinal mucosa and increase the risk of death (4). Therefore, there is an urgent need to identify safe and effective feed additives to reduce immune stress.

Chlorogenic acid (CGA), a natural compound derived from plants, offers several advantages as a feed additive (5). It is a potent antioxidant that helps restore the balance of oxidation-reduction states within cells (6). Studies have demonstrated that CGA has a good safety profile, with no significant adverse reactions or toxicity to normal cells and tissues (7). CGA exhibits immunomodulatory effects, promotes growth and development by regulating immune system activity, reduces disease incidence, and alleviates stress responses (8, 9). CGA supplementation can improve growth performance, intestinal barrier function, and oxidation-reduction status, and it has also been found effective in preventing intestinal inflammation and barrier damage induced by dexamethasone (10).

The gut microbiota is a complex, dynamic ecosystem in which bacterial populations interact with the host as well as each other and play a critical role in shaping tissue and organ morphogenesis, immune responses, metabolic processes, and overall host health. The host provides the growth environment for beneficial gut microbes, which, in turn, help to maintain homeostasis (11). The gut microbiota plays a crucial role in shaping the host’s immune system and enhancing resistance by influencing gut morphology, feed digestion and nutrient absorption, immune system development, and the optimal balance of beneficial gut microbes (12, 13). The microbial community produces large amounts of metabolites such as fatty acids, lipids, amino acids, and short peptides in response to environmental stimuli. These metabolites play a crucial role in the maturation and maintenance of the normal functioning of the host immune system (14). Previous studies have shown that CGA can regulate the composition of the intestinal microbiome by increasing the proportion of bacteria that produce short-chain fatty acids (SCFAs) and reducing the production of intestinal toxins (15, 16). The action of CGA can reduce the harmful effects of an overactive inflammatory response, significantly improve gut dysbiosis, increase the abundance of beneficial bacteria, inhibit the growth of harmful bacteria, and maintain intestinal homeostasis (17). Rectal administration of CGA can alleviate post-infection irritable bowel syndrome (PI-IBS) by regulating the intestinal microbiome and its metabolites (18). Supplementation of CGA in immune-stressed broilers can regulate intestinal flora and increase serum beneficial metabolites, thereby improving intestinal health and immune function (10). Our previous research indicated that CGA could effectively alleviate immune stress in broilers; however, it was unclear whether its antistress effects were achieved by altering the gut microbiota and metabolites in the ileum of immune-stressed broilers. Therefore, in this study, we established an immune stress model and used 16S rRNA gene sequencing and metabolomics technology with mass spectrometry analysis to investigate the impacts of dietary supplementation with 1,000 mg/kg CGA on the composition of ileal microbiota, metabolite profiles, and intestinal health in immune-stressed broilers, to elucidate its mechanism in relieving stress through microbiota-metabolite interactions.

## RESULTS

### Diversity of the ileal microbiota

**Fig 1:**
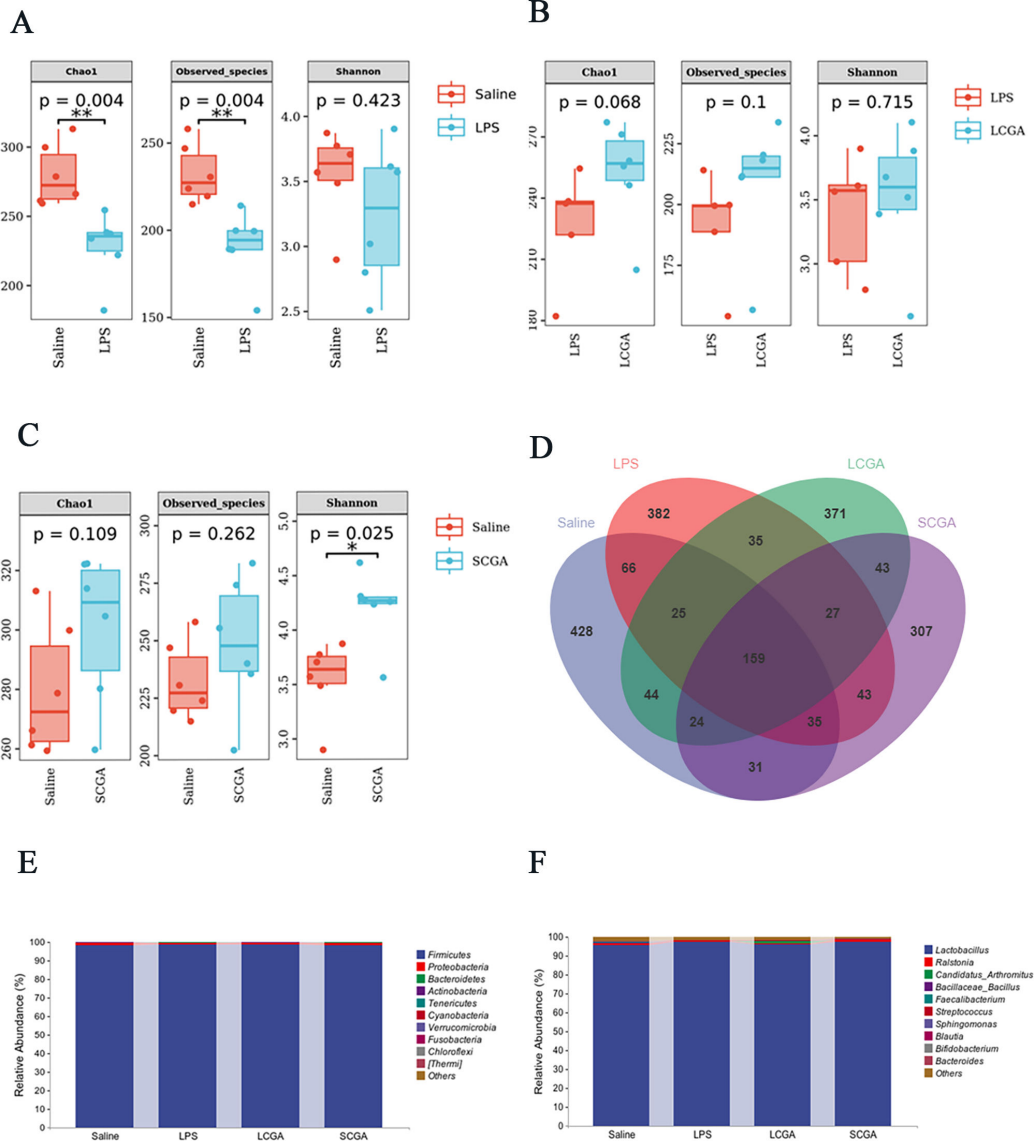
Diversity of microbiota in the ileum of broilers in different treatments. Alpha diversity in (**A**) Saline vs LPS, (**B**) LPS vs LCGA, and (**C**) Saline vs SCGA. (**D**) Venn diagram. (**E**) Bacterial composition at the phylum level in the ileum. (**F**) Bacterial composition at the genus level in the ileum.

On average, 84,747 high-quality sequences were obtained from each of the 24 ileal content samples. The α diversity showed that compared with the saline group, the Chao1 index and the observed species index of the LPS group were significantly lower, and the Shannon index of the SCGA group was significantly higher (*P* < 0.05) (Fig. 1A through C).

### Composition of the ileal microbiota

**Fig 2:**
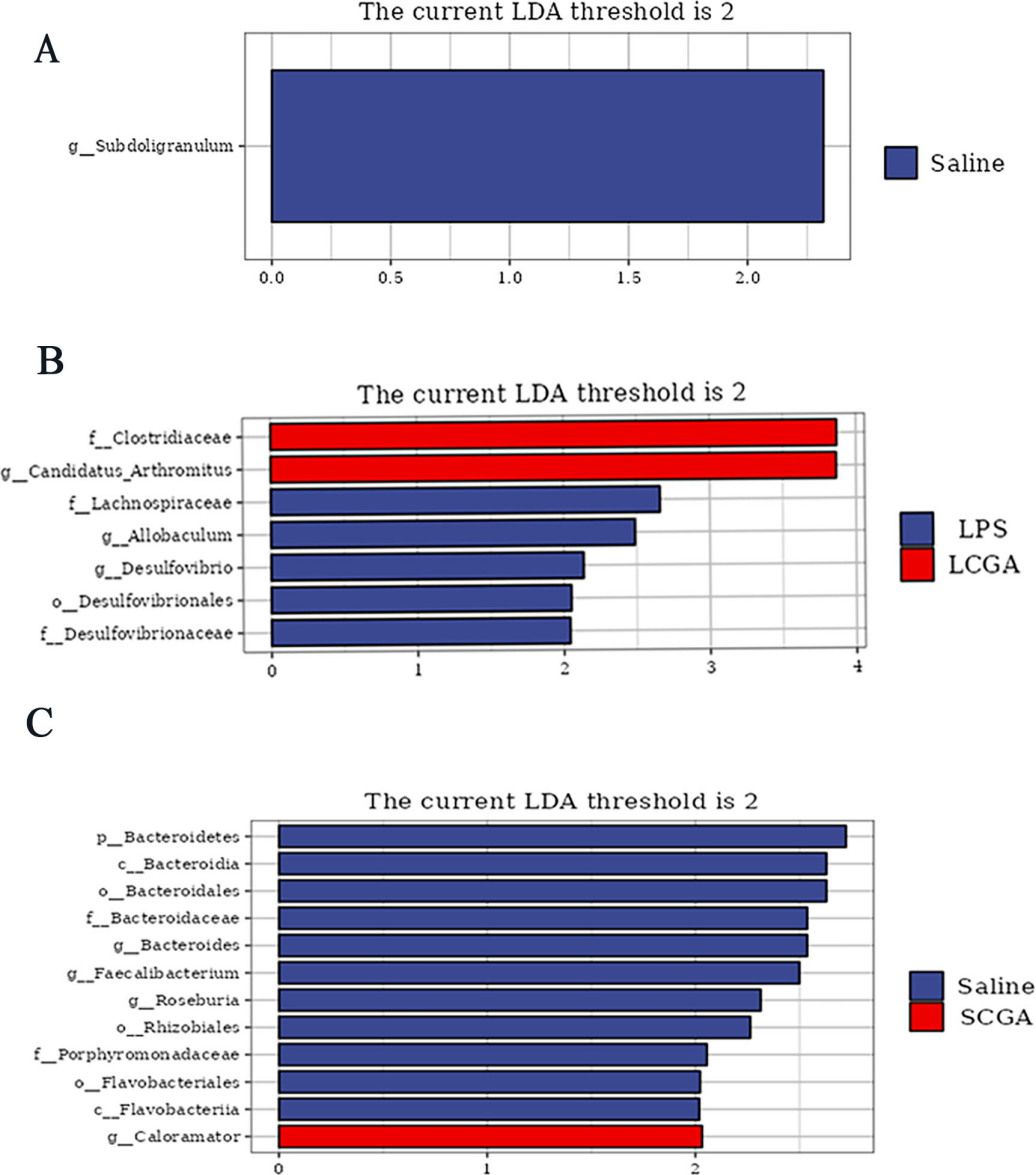
Histogram of LDA value distribution between Saline and LPS groups (**A**), LPS and LCGA groups (**B**), Saline and SCGA groups (**C**). Biomarkers were identified by linear discriminant analysis coupled with effect size (LEfSe) using the default parameters.

The Venn diagram (Fig. 1D) showed that there are 159 OTUs in common among all groups, while there are 428, 382, 371, and 307 unique OTUs in the saline, LPS, LCGA, and SCGA groups, respectively. The microbial species composition analysis showed that the dominant phyla in the four groups are *Firmicutes*, *Proteobacteria*, and *Bacteroidetes* (Fig. 1E); *Bacteroidetes* was significantly lower in the LPS group compared with the saline group (*P* < 0.05). At the genus level (Fig. 1F), the dominant genera in the four groups were *Lactobacillus*, *Ralstonia*, and *Candidatus_Arthromitus*, with no significant differences among the four groups (*P* > 0.05). The LEfSe analysis showed that the relative abundance of *Subdoligranulum* in the LPS group was significantly lower than that in the saline group (*P* < 0.05) (Fig. 2A). Compared with the LPS group, the relative abundance of *Clostridiaceae* and *Candidatus Arthromitus* in the LCGA group was higher, while the relative abundance of *Lachnospiraceae*, *Allobaculum*, *Desulfovibrio*, *Desulfovibrionales*, and *Desulfovibrionaceae* was lower (*P* < 0.05) (Fig. 2B). Compared with the saline group, the relative abundance of *Caloramator* in the SCGA group was higher, while the relative abundances of *Bacteroidetes*, *Bacteroidia*, *Bacteroidales*, *Bacteroidaceae*, *Bacteroides*, *Faecalibacterium*, *Roseburia*, *Rhizobiales*, *Porphyromonadaceae*, *Flavobacteriales*, and *Flavobacteriia* were lower (*P* < 0.05) (Fig. 2C).

### Ileal metabolites and metabolic pathways

**Fig 3:**
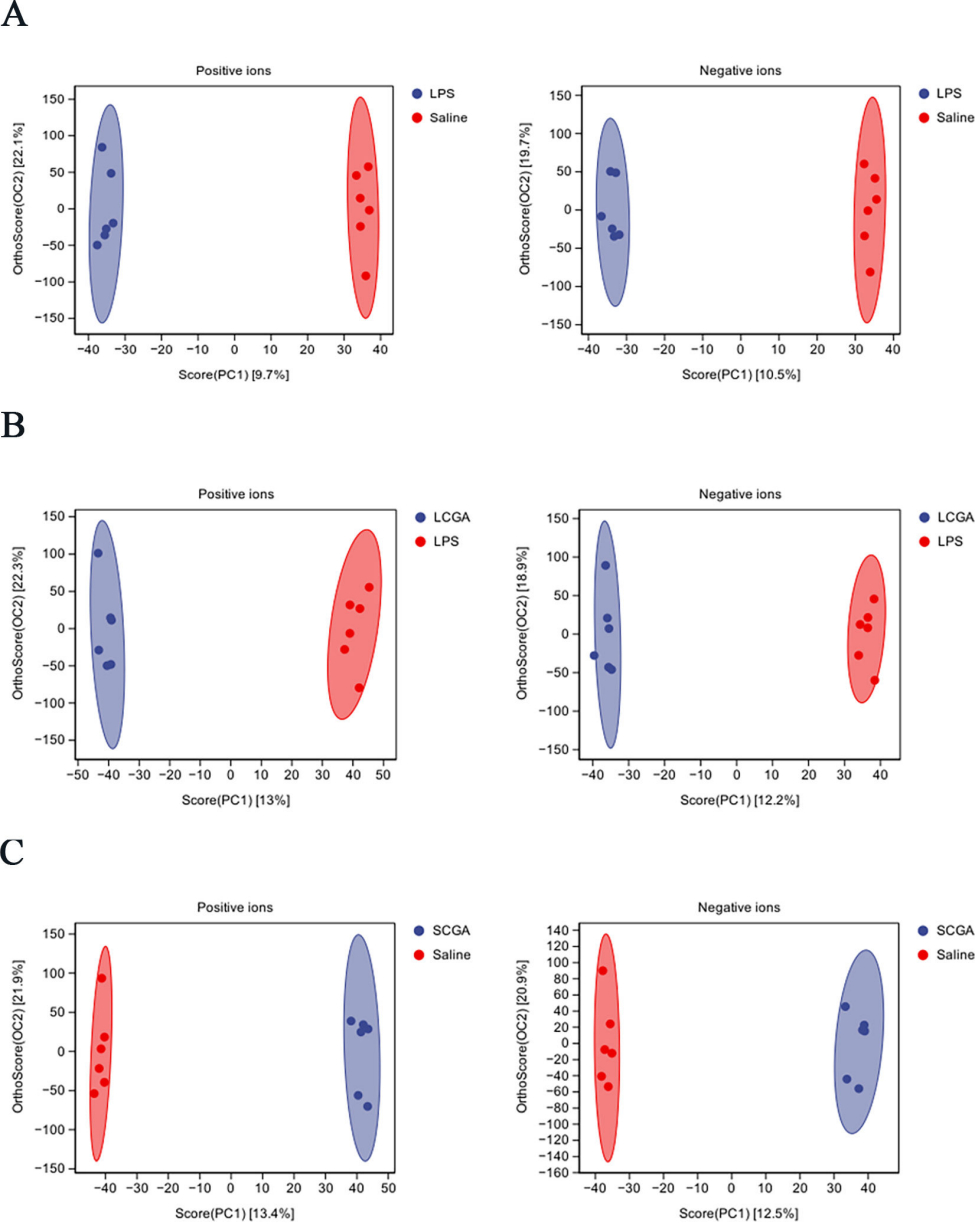
OPLS-DA score plots for positive and negative ions. OPLS-DA score plot of (**A**) Saline vs LPS, (**B**) LPS vs LCGA, and (**C**) Saline vs SCGA. PC1 is principal component 1, PC2 represents principal component 2, and each point is one sample.

**Fig 4:**
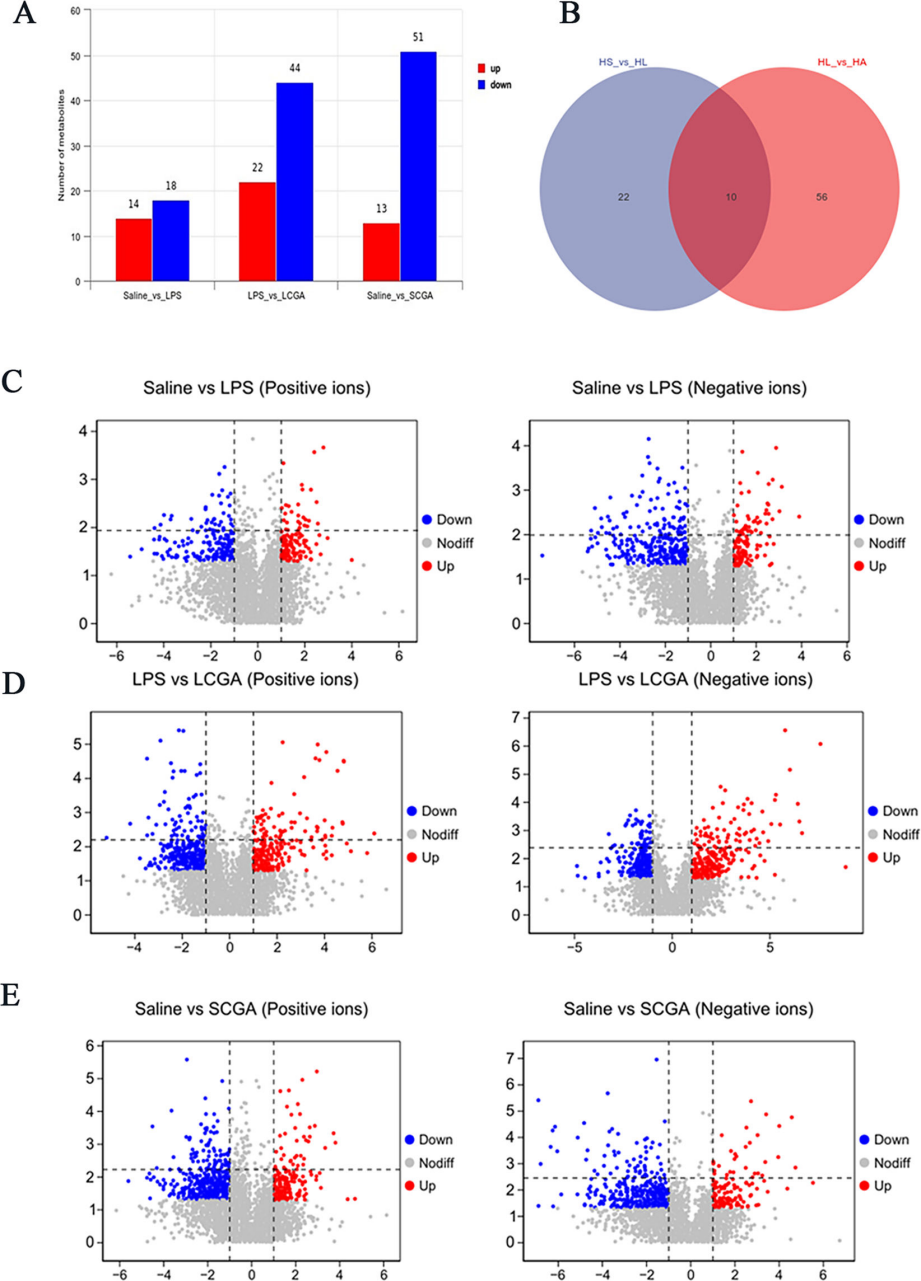
Significant differentially expressed metabolites in the ileum of broilers in different groups. (**A**) Differential metabolite histogram. (**B**) Venn diagram. Volcano plots of (**C**) Saline vs LPS, (**D**) LPS vs LCGA, and (**E**) Saline vs SCGA.

Clear separations were observed in the OPLS-DA score plots (Fig. 3A through C). Figure 4A shows the number of differentially expressed metabolites (DEMs) in each group. Compared with the saline group, 32 DEMs were found in the LPS group, with 14 upregulated and 18 downregulated (*P* < 0.05). Compared with the LPS group, 66 DEMs were found in the LCGA group, with 22 upregulated and 44 downregulated (*P* < 0.05). Compared with the saline group, 64 DEMs were found in the SCGA group, with 13 upregulated and 51 downregulated (*P* < 0.05). Two comparison groups identified 10 common DEMs (Fig. 4B). The changes in metabolite profiles are shown in Fig. 4C through E, and the specific DEMs are listed in Tables 1 and 2. Compared with the saline group, pyroglutamic acid, L-glutamic gamma-semialdehyde, and biliverdin were downregulated in the LPS group, while trans-ferulic acid, methylmalonic acid, pantothenic acid, and N-acetyl-L-aspartate were upregulated. Compared with the LPS group, corticosterone, prostaglandin B2, trans-ferulic acid, and methylmalonic acid were downregulated in the LCGA group, while pyroglutamic acid, L-glutamic gamma-semialdehyde, biliverdin, chenodeoxycholic acid, desmosterol, and 4-hydroxycinnamic acid were upregulated (*P* < 0.05).

**TABLE 1:** Differences of metabolites in the LPS group compared with the Saline group

ID	Name	*P* value	VIP	Regulation
M131T54_2.pos	L-Glutamic gamma-semialdehyde	0.002	2.689	Down
M130T54.pos	Pyroglutamic acid	0.003	2.647	Down
M583T420_1.pos	Biliverdin	0.007	2.367	Down
M101T688.pos	Methylmalonic acid	0.015	2.174	Up
M193T90.neg	trans-Ferulic acid	0.023	2.036	Up
M174T45.neg	N-Acetyl-L-aspartic acid	0.046	1.837	Up
M218T56.neg	Pantothenic acid	0.039	1.835	Up

**TABLE 2:** Differences of metabolites in the LCGA group compared with the LPS group

ID	Name	*P* value	VIP	Regulation
M101T688.pos	Methylmalonic acid	0.013	1.931	Down
M163T77.neg	4-Hydroxycinnamic acid	0.000	2.605	Up
M391T446_1.neg	Chenodeoxycholic acid	0.003	2.222	Up
M583T420_1.pos	Biliverdin	0.008	2.082	Up
M193T90.neg	trans-Ferulic acid	0.009	2.042	Down
M131T54_2.pos	L-Glutamic gamma-semialdehyde	0.009	2.005	Up
M329T482.pos	Corticosterone	0.005	2.000	Down
M384T463.pos	Desmosterol	0.011	1.978	Up
M130T54.pos	Pyroglutamic acid	0.028	1.762	Up
M317T533.pos	Prostaglandin B2	0.049	1.708	Down

**Fig 5:**
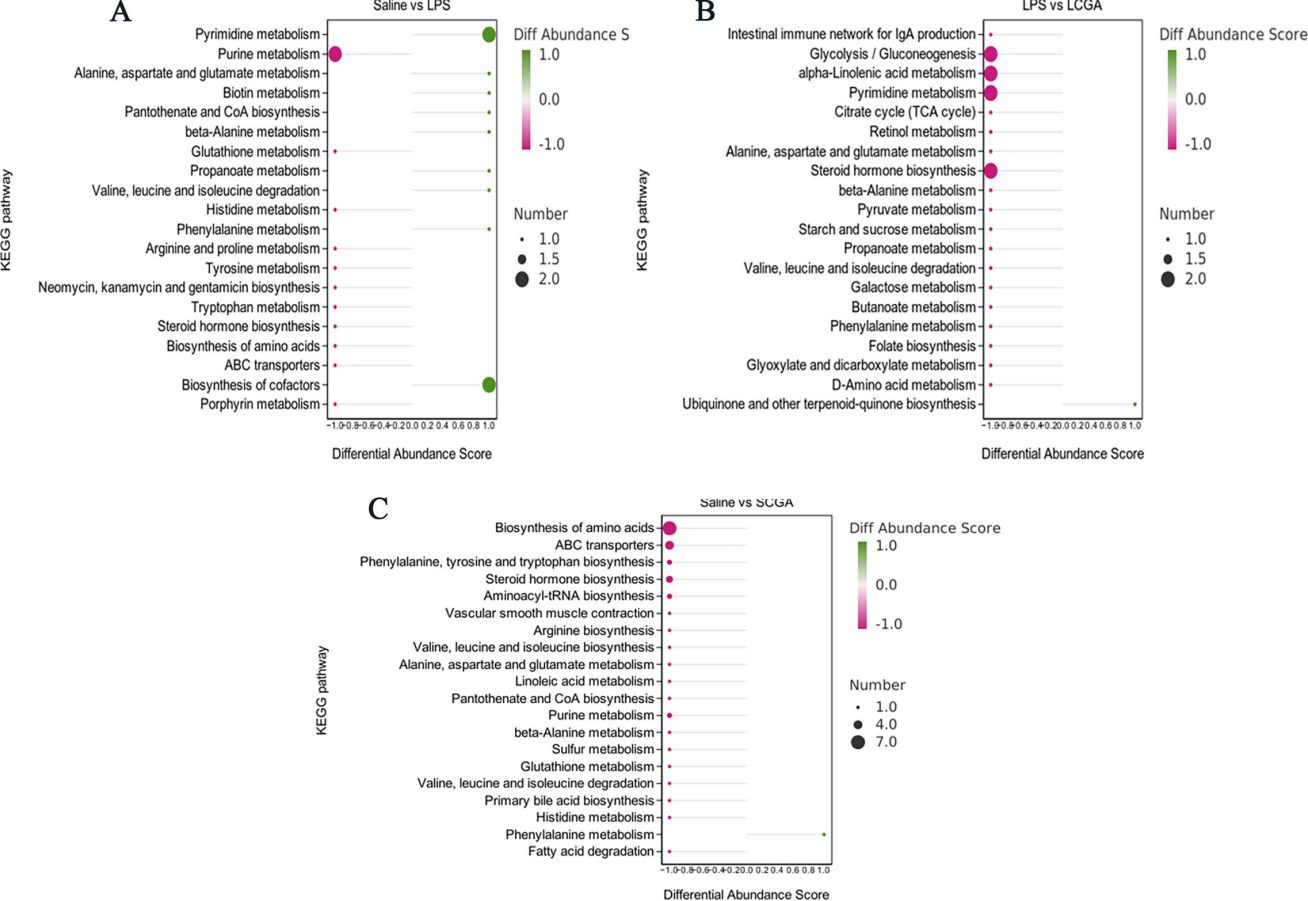
Differential metabolic pathways in the ileum of broilers in different treatment groups. KEGG pathway enrichment diagram of (**A**) Saline vs LPS, (**B**) LPS vs LCGA, and (**C**) Saline vs SCGA.

To comprehensively evaluate the metabolic pathways in the ileum of broilers, DEMs were subjected to KEGG enrichment analysis (Fig. 5A through C). Compared with the saline group, the differential metabolic pathways in the ileum of the LPS group mainly centered on pyrimidine metabolism (*P* < 0.05). Compared with the LPS group, the differential metabolic pathways in the ileum of the LCGA group mainly included five pathways: pentose phosphate pathway, tyrosine metabolism, ABC transporters, intestinal immune network for IgA production, and glycolysis/gluconeogenesis (*P* < 0.05). Compared with the saline group, the differential metabolic pathways in the ileum of the SCGA group mainly included eight pathways: biosynthesis of amino acids, cysteine and methionine metabolism, tryptophan metabolism, neuroactive ligand-receptor interactions, steroid biosynthesis, arachidonic acid metabolism, ABC transporters, phenylalanine, tyrosine and tryptophan biosynthesis (*P* < 0.05).

### Combined analysis of microbiome and metabolome

**Fig 6:**
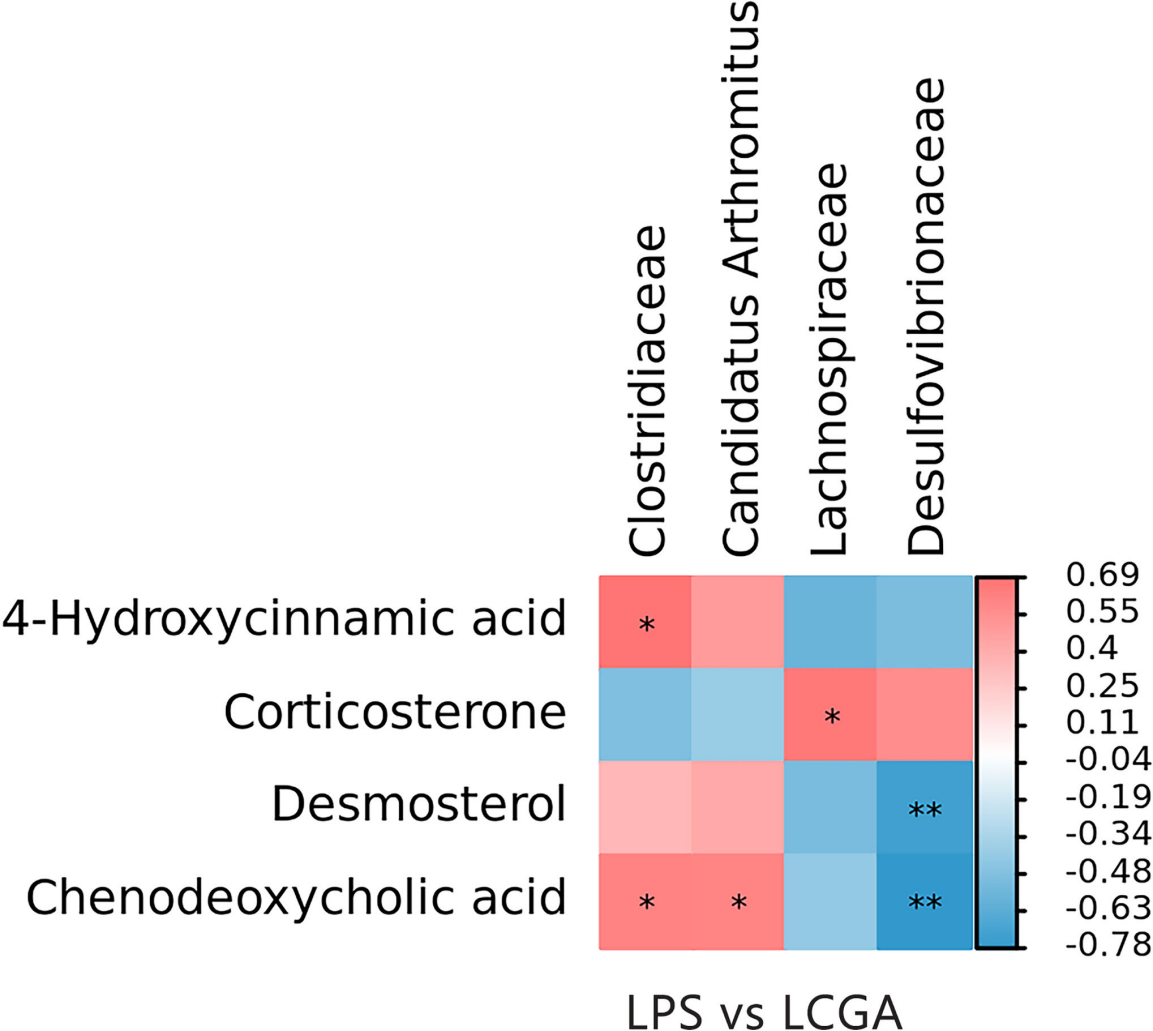
Correlations between the different microbiota and metabolites in the ileum of LPS and LCGA groups. The color corresponds to the Spearman rank correlation coefficient distribution. Red represents a significant positive correlation, while blue represents a significant negative correlation. The intensity of the color represents the strength of the correlation. * representing *P* < 0.05, ** representing *P* < 0.01.

Spearman’s rank correlation coefficient was employed to examine the association between the ileal microbiome and the metabolites identified by mass spectrometry. The study found that *Lachnospiraceae* was positively correlated with corticosterone (*P* < 0.05). *Clostridiaceae* was positively correlated with chenodeoxycholic acid and 4-hydroxycinnamic acid, while *Candidatus Arthromitus* was positively correlated with chenodeoxycholic acid (*P* < 0.05). *Desulfovibrionaceae* was negatively correlated with chenodeoxycholic acid and desmosterol (*P* < 0.05) (Fig. 6).

## DISCUSSION

The gut microbiota play an important role in maintaining host health, immunity, and metabolism (19). In the current study, the Chao1 index and the observed species were significantly lower in the LPS group compared with the saline group, and the Shannon index was significantly higher in the SCGA group compared to the saline group, suggesting that immune stress caused a decrease in microbial diversity, and supplemented normal broilers with CGA enhanced microbial diversity. This is consistent with our previous finding (20). LEfSe analysis showed a significant decrease in the relative abundance of *Subdoligranulum* after LPS injection. This is a genus of butyric acid-producing bacteria, which are important for the maintenance of intestinal health and regulation of immune function (21). Butyric acid is involved in several important physiological functions such as amelioration of mucosal inflammation, attenuation of oxidative stress, strengthening of the epithelial barrier, and prevention of colorectal cancer (22). It has been shown that oxidative stress causes a decrease in the abundance of *Subdoligranulum* in the cecum of laying hens (23). And there is a positive correlation between the abundance of *Subdoligranulum* and microbial richness (24). In agreement with our results, other studies have shown that the abundance of *Subdoligranulum* in the gut is significantly reduced in patients with irritable bowel syndrome (25). These results showed that immune stress significantly decreased the abundance of *Subdoligranulum*, which led to an imbalance in the intestinal microflora and poor health of broilers.

Broiler intestines are susceptible to infection by enteric bacteria, and the microbial community plays an important role in the intestinal defense system (26, 27). It has been demonstrated that CGA can regulate the gut microbiota and maintain the homeostasis of the gut (28). In this research, the supplementation of chlorogenic acid significantly increased the relative abundance of *Clostridiaceae*, *Candidatus Arthromitus*, and significantly decreased the relative abundance of *Lachnospiraceae, Allobaculum, Desulfovibrionaceae, Desulfovibrio*, and *Desulfovibrionales. Clostridiaceae*, along with other probiotic bacteria that maintain the intestinal microecological balance, reduce the growth of potential pathogens, and promote intestinal health (29). *Candidatus Arthromitus* bacteria share characteristics of the *Clostridiaceae* family and are a type of gut commensal bacteria capable of promoting adaptive and innate immune responses in mice, preventing diseases, and facilitating animal growth (30). They also play a role in maintaining gut homeostasis, which has a positive effect on the immune function of broiler chickens (31, 32). Studies have shown that their colonization in the intestines is beneficial to the host by inhibiting the growth of pathogens and preventing viral infections (33, 34). The above results indicate that dietary supplementation with CGA can enhance the level of beneficial microbiota, such as *Clostridiaceae* and *Candidatus Arthromitus*, to maintain the balance of intestinal immune function. It has been proven that *Lachnospiraceae* can enhance the intestinal barrier by producing short-chain fatty acids and participate in regulating the host’s immune system and energy metabolism (35). Interestingly, in some individuals with inflammatory bowel disease (IBD), the abundance of *Lachnospiraceae* showed an atypical increase (36). This change does not necessarily signify harmful effects on the host but may be related to changes in the intestinal environment caused by the disease itself or the related treatments. Wang (17) reported that CGA reversed the intestinal microbiota dysbiosis induced by a high-fat diet (HFD) through the inhibition of *Lachnospiraceae* and *Desulfovibrionaceae*. Some bacterial species in the *Desulfovibrionales* and *Desulfovibrionaceae* can reduce sulfates to produce toxic H_2_S, which can damage the intestinal mucus barrier by breaking disulfide bonds, exposing the epithelium to bacteria and toxins, and ultimately leading to inflammation (37). *Allobaculum* has been positively linked to susceptibility to autoimmune encephalitis (38). Results of our study suggest that CGA can restore the balance of gut microbiota caused by immune stress by increasing the abundance of beneficial bacteria like *Clostridiaceae* and *Candidatus Arthromitus*, and inhibiting *Lachnospiraceae, Allobaculum, Desulfovibrio, Desulfovibrionales,* and *Desulfovibrionaceae*.

Our results show that immune stress leads to the downregulation of metabolites, such as pyroglutamic acid, L-glutamic gamma-semialdehyde, biliverdin, and the upregulation of trans-ferulic acid, while adding CGA to the diet can reverse these metabolic changes. During immune stress, the body increases the rate of protein synthesis and degradation to provide amino acids to support the energy needs of immune cells (39). Pyroglutamic acid and L-glutamic gamma-semialdehyde are derivatives of glutamic acid, an important precursor for glutathione, which protects cells from oxidative stress (40). In this study, CGA significantly increased glutamate and L-glutamic gamma-semialdehyde, thereby enhancing cellular antioxidant capacity, which is consistent with our previous research (20). Biliverdin has antioxidant properties and provides effective protection against oxidative stress both *in vivo* and *in vitro* (41). It can inhibit the adhesion of white blood cells by downregulating the expression of selectin in inflammation models (42). Studies have shown that trans-ferulic acid (TFA) reduces the levels of reactive oxygen species (ROS) and nitric oxide (NO) in cells induced by high blood sugar, while increasing the level of glutathione (GSH) (43). The upregulation of TFA may be part of the body’s attempt to suppress oxidative stress damage by inhibiting the release of oxidative factors and increasing the production of antioxidants. Adding CGA to the diet can significantly upregulate biliverdin and downregulate trans-ferulic acid. Our results indicate that immune stress downregulated pyroglutamic acid, L-glutamic gamma-semialdehyde, and biliverdin and upregulated TFA, which reduced antioxidant capacity and caused intestinal oxidative damage and inflammation. However, CGA can reverse these metabolic changes and restore antioxidant capacity, protecting the body from oxidative damage.

Immune stress increased the levels of methylmalonic acid, pantothenic acid, and N-acetyl-L-aspartic acid and decreased pyroglutamic acid and L-glutamic gamma-semialdehyde, but the addition of CGA to broiler feed reversed these effects. The tricarboxylic acid cycle is the center of energy production in the intestine (44). Under normal conditions, methylmalonic acid (MMA) enters the TCA cycle through propionyl CoA and methylmalonyl-CoA to generate a large amount of ATP, but high levels of MMA may inhibit mitochondrial energy metabolism in tissues and biological fluids (45). MMA is a byproduct of mitochondrial metabolism, and its accumulation is closely related to mitochondrial dysfunction and energy metabolism disorder (46). Under immunologic stress, the increased energy demand leads to an overload of the tricarboxylic acid (TCA) cycle, resulting in enhanced MMA production (47). Elevated levels of MMA can inhibit mitochondrial enzyme activity, impair ATP synthesis, and exacerbate oxidative stress and inflammatory responses (48). In our experiments, CGA reversed the accumulation of methylmalonic acid caused by immune stress. Pantothenic acid not only participates in coenzyme A synthesis for energy production via the TCA cycle but also functions in the synthesis of N-acetyl-L-aspartate (NAA) (49). Studies have shown that supplementation with NAA can increase the level of α-ketoglutarate, which is an intermediate in the TCA cycle (50). Pyroglutamic acid and L-glutamic gamma-semialdehyde are derivatives of glutamic acid, which can be converted into α-ketoglutarate by the action of glutamate dehydrogenase and enter the TCA cycle to produce energy (40). There is a significant decrease in N-acetyl-L-aspartate and significant increases in pyroglutamic acid and L-glutamic gamma-semialdehyde in the ileum of immune-stressed broilers after CGA treatment. These results suggest that immune stress can disrupt normal energy metabolism in broiler chickens, with a large proportion of the energy resources being diverted from growth and development to respond to stress. Dietary addition of CGA reversed this energy metabolism switch.

Dietary CGA addition significantly downregulated prostaglandin B2 and corticosterone in the ileum of immune-stressed broiler chickens, as well as upregulating chenodeoxycholic acid, desmosterol, and 4-hydroxycinnamic acid. Prostaglandin B2 is a metabolite of the arachidonic acid metabolic pathway, obtained by dehydration from prostaglandin E2 (PGE2), and plays a key role in regulating inflammatory responses (51, 52). Corticosterone, which activates the hypothalamic-pituitary-adrenal axis (HPA) and stimulates the adrenal gland to produce large amounts of inflammatory cytokines, is upregulated in chickens under stress and positively correlates with their stress level (53). Adding CGA to the diet significantly reduced prostaglandin B2 and corticosterone levels in the ileum of immune-stressed broiler chickens, thereby alleviating stress and reducing the inflammatory response. Chenodeoxycholic acid is one of the primary bile acids, and studies have shown that supplementation with chenodeoxycholic acid can activate the farnesol X receptor (FXR), suppress pro-inflammatory cascades like NF-κB, curtail the liberation of inflammatory mediators, alleviate oxidative stress, and enhance both gut microbiota diversity and immune competence (54). It has also been reported that a reduction in mitochondrial desmosterol promoted the production of ROS and the activation of NLRP3-dependent inflammasomes in macrophages (55). Increasing the expression of desmosterol improved experimental peritonitis and non-alcoholic steatohepatitis (56). The phenolic compound, 4-hydroxycinnamic acid, protects cells from mitochondrial dysfunction by maintaining redox homeostasis and improving mitochondrial membrane potential (57). The above results support the hypothesis that CGA reduces LPS-induced intestinal damage by downregulating prostaglandin B2 and corticosterone and upregulating chenodeoxycholic acid, 4-hydroxycinnamic acid, and desmosterol, thereby increasing the expression of anti-inflammatory factors and antioxidant capacity.

In this study, Spearman’s correlation analysis revealed that bacteria of the family *Lachnospiraceae* displayed a significant positive correlation with corticosterone concentration. In mice, the intestinal bacteria influence the HPA axis, regulating the production of corticosterone (58). In other studies, a positive correlation was observed between *Lachnospiraceae* and stress levels in the water-immersion restraint stress (WIRS) mouse model (59). Our results showed that the abundance of *Clostridiaceae* in the gut was positively correlated with chenodeoxycholic acid and 4-hydroxycinnamic acid levels, while *Candidatus Arthromitus* was significantly positively correlated with chenodeoxycholic acid. Some studies have demonstrated that *Lachnospiraceae* and *Clostridiaceae* are capable of producing reactive sulfur species (RSS), which can suppress oxidative damage and enhance antioxidant capacity (60, 61). Chenodeoxycholic acid is positively correlated with gut microbiota diversity, and a number of *Clostridium* species can synthesize chenodeoxycholic acid (62). The increased abundance of *Clostridiaceae* and *Candidatus Arthromitus* among the intestinal microbes of broilers and their synthesis of chenodeoxycholic acid and 4-hydroxycinnamic acid significantly enhanced antioxidant capacity and inhibited oxidative damage to the body. By contrast, there was a significant negative correlation between the presence of *Desulfovibrionaceae* in the gut microbial community and the levels of chenodeoxycholic acid and desmosterol. Members of the *Desulfovibrionaceae* family are associated with increased inflammation. Correlation analysis suggests that CGA exerts anti-inflammatory, antioxidant, and mitochondrial function-enhancing effects by modulating the intestinal microbiota, including *Lachnospiraceae*, *Clostridiaceae*, *Candidatus Arthromitus*, and *Desulfovibrionaceae*, as well as their associated metabolites, corticosterone, chenodeoxycholic acid, 4-hydroxycinnamic acid, and desmosterol.

In summary, CGA can significantly increase the abundance of beneficial bacteria, such as members of the *Clostridiaceae* and *Candidatus Arthromitus* in the ileum of immune-stressed broilers, reduce the abundance of pathogenic bacteria, and enhance intestinal immune function. CGA supplementation leads to a significant downregulation of corticosterone and prostaglandin B2 in ileal metabolites of immune-stressed broilers, as well as a significant upregulation of pyroglutamic acid, L-glutamic gamma-semialdehyde, biliverdin, chenodeoxycholic acid, desmosterol, and 4-hydroxycinnamic acid, which indicates that CGA can enhance antioxidant capacity and alleviate inflammatory responses induced by immune stress. These changes result in a significant improvement in antioxidant capacity and alleviation of inflammatory responses induced by immune stress, thereby exerting beneficial effects on broilers.

## MATERIALS AND METHODS

### Animals and experimental conditions

Healthy 1-day-old male Arbor Acres broiler chickens were obtained from QuanDa Poultry Breeding Co., Ltd. (Luo Yang, China). All broilers were raised in an environment maintained at constant temperature and humidity with adequate ventilation. A light/dark cycle was employed, with continuous light for the first 3 days followed by 23 hours of light and 1 hour of darkness. During the first week, the room temperature was maintained at 33°C ± 1°C and was subsequently reduced by 2°C–3°C per week until it reached 25°C. This temperature was maintained until euthanasia. Throughout the experiment, the environmental humidity was controlled at 40%–60%. All broilers had *ad libitum* access to feed and water.

### Experimental design

The experiments involved 312 broilers, randomly divided into four treatment groups, with six replicates in each group and 13 broilers per replicate. The groups were as follows: (1) Saline, injected with saline and fed a basal diet; (2) LPS, injected i.p. with 0.5 mg/kg LPS and fed a basal diet; (3) LCGA, injected with 0.5 mg/kg LPS and fed a basal diet supplemented with 1,000 mg/kg CGA; and (4) SCGA, injected with saline and fed a basal diet supplemented with 1,000 mg/kg CGA. The LPS and LCGA groups were intraperitoneally injected with 0.5 mg/kg LPS on days 14, 15, and 16 to induce immune stress. The Saline and SCGA groups were intraperitoneally injected with the same dose of saline. The dosages of LPS and CGA were determined based on previously experimental results (20, 63–65). The formulation of the basal diet complied with the requirements of the National Research Council, and the feed ingredients were consistent with those used in previous experiments (63). CGA (HPLC ≥98%) was purchased from Changsha Staherb Natural Ingredients Co., Ltd (Changsha, China). Lipopolysaccharide (LPS) was obtained from Sigma-Aldrich Trading Co., Ltd (Shanghai, China). The serotype of *Escherichia coli* used was O55:B5.

### Sample collection

At 17 days of age, one chicken was randomly selected from each replicate and euthanized by cervical dislocation, after which an intestinal segment and its contents were quickly removed into a 2 mL sterile centrifuge tube, rapidly frozen in liquid nitrogen, and stored at −80°C for subsequent analysis of microbiome and non-targeted metabolomics analysis.

### Ileal microbiome analysis

From each group, six samples of the ileal contents were obtained and sent to the Shanghai Personal Biotechnology Co., Ltd. for microbiome analysis. Bacterial DNA was extracted using a kit, and the DNA concentration was determined with a microvolume ultraviolet spectrophotometer. The target DNA was amplified by PCR, and the PCR products were verified by agarose gel electrophoresis. The community DNA fragments were sequenced on an Illumina platform in a paired-end manner. The raw data set from the high-throughput sequencing was screened for sequence quality, and the raw screened sequences were divided into libraries and samples according to index and barcode information, and the barcode sequences were removed. The sequences were denoised or OTU clustered according to the dada2 analysis flow of QIIME2 or the Vsearch software analysis flow. The specific composition of each sample at different taxonomic levels was displayed, and alpha diversity was calculated using the unnormalized ASV/OTU table. The LEfSe analysis was conducted using the Kruskal-Wallis rank sum test, Wilcoxon rank sum test, and linear discriminant analysis (LDA) to find the signature species. The metabolic functions of the samples were predicted based on the 16S rRNA gene sequencing results, and the differences in pathways were identified, with the species composition of specific pathways obtained.

### Non-targeted metabolomics analysis of ileal metabolites

Six samples from each group were sent to Shanghai Personal Biotechnology Co., Ltd for metabolomics analysis. First, metabolites were extracted from the samples, and an aliquot of the extracted samples was mixed to form a QC sample, which was analyzed using LC-MS/MS technology (66). The mass spectrometry data were preprocessed, including peak identification, noise removal, and baseline correction. Metabolites in biological samples were identified by comparing the mass spectrometry data with databases of known fragment patterns. Differential metabolite screening and KEGG enrichment analyses were performed using unit statistical analysis and multivariate statistical analysis for all metabolites detected by MS/MS in both positive and negative ion modes (*P* < 0.05, VIP >1).

### Statistical analysis

All data were analyzed using one-way ANOVA in SPSS 20.0. A *P* value of < 0.05 was considered statistically significant. A correlation matrix between gut microbiota and metabolites was generated using Spearman’s rank correlation coefficient. Visualization of hierarchical clustering by heatmaps and correlation matrices was achieved using the R language.

## Data Availability

The data presented in the study are deposited in NCBI under accession number PRJNA1188052 and Metabolights under accession number MTBLS11790.
